# Selective stimulation of rat sciatic nerve using an array of mm-size magnetic coils: a simulation study

**DOI:** 10.1049/htl.2018.5020

**Published:** 2019-06-03

**Authors:** Pragya Kosta, David J. Warren, Gianluca Lazzi

**Affiliations:** 1 Department of Electrical and Computer Engineering, University of Utah, Salt Lake City, UT 84112, USA; 2 Department of Biomedical Engineering, University of Utah, Salt Lake City, UT 84112, USA; 3 Department of Electrical Engineering, University of Southern California, Los Angeles, CA 90089, USA; 4 Department of Ophthalmology, University of Southern California, Los Angeles, CA 90033, USA

**Keywords:** biological tissues, physiological models, neurophysiology, neuromuscular stimulation, bioelectric potentials, muscle, biomagnetism, bioelectric phenomena, biomedical electrodes, targeted region, nontargeted regions, selectivity index, array configuration, traditional extraneural electrode arrays, selective neurostimulation, selective stimulation, rat sciatic nerve, mm-size magnetic coils, magnetic neural stimulation, electrical stimulation, selective activation, selective neural stimulation, single coil, three-dimensional heterogeneous multiresolution nerve model, magnetic fields, electric fields, performance metric

## Abstract

This work proposes and computationally investigate the use of magnetic neural stimulation as an alternative to electrical stimulation to achieve selective activation of rat sciatic nerve. In particular, they assess the effectiveness of an array of small coils to obtain selective neural stimulation, as compared to a single coil. Specifically, an array of four mm-sized coils is used to stimulate rat sciatic nerve, targeting the regions of fascicles that are associated with different muscles of the leg. To evaluate the selectivity of activation, a three-dimensional heterogeneous multi-resolution nerve model is implemented using the impedance method for the computation of the magnetic and electric fields in the nerve. The performance metric ‘selectivity index’ is defined that measures the recruitment of the targeted region compared to other non-targeted regions of the nerve. The selectivity index takes values between −1 (least selective) and 1 (most selective). For each targeted region, a selectivity index of 0.75 or better is predicted for the proposed array configuration. The results suggest that an array of coils can provide superior spatial control of the electric field induced in the neural tissue compared to traditional extraneural electrode arrays, thus opening the possibility to applications where selective neurostimulation is of interest.

## 1 Introduction

Magnetic neural stimulation has received considerable interest as an alternative to electrical neurostimulation for both central nervous system and peripheral nervous system (PNS) neurostimulation applications [1–4]. Despite lower energy requirements, electrical neural stimulation has drawbacks compared to magnetic stimulation, including the need for direct contact between tissue and electrodes and limited control of patterns of fields induced in the neural tissue. Direct contact between tissue and electrode surfaces leads to electrode corrosion and tissue damage [3], and to recruitment of an immune response. On the contrary, magnetic stimulation can elicit neural activity at the proximal tissue without direct contact between coils and neural tissue. That is, metal coils can be completely insulated in biocompatible materials. Further, field patterns can be controlled because the tissue has uniform relative magnetic permeability. However, the energy requirement can be significantly higher compared to electrical stimulation [3–5].

Central and peripheral neural tissues (e.g., cerebral cortex and sciatic nerve) are highly structured [6]. In the PNS, motor fibres are grouped together depending on the muscle that they innervate. To recreate natural movement, the selective stimulation of the appropriate fibre groups of the nerve, without inadvertently activating surrounding fibre groups, is essential. Various extraneural and intraneural interfaces have been developed for selective peripheral nerve stimulation [7–10]. Intraneural electrodes, such as Transversal Intrafascicular Multichannel Electrode and Longitudinal IntraFascicular Electrode, penetrate the epineurium. On the other hand, extraneural electrodes are placed outside the epineurium. As the intraneural electrodes reach closer to the stimulation site, they achieve higher stimulation selectivity at the expense of higher invasiveness. The foreign body reaction causes an encapsulation around the tissue-electrode interface which leads to gradual signal decay and stimulation threshold increment [10]. Even though the techniques to improve interneural stimulation are rapidly increasing; at present, the extraneural stimulation is regarded as a more suitable option for long-term implantation [9, 11]. Examples of extraneural electrodes are multichannel cuff electrode and Flat Interface Nerve Electrode (FINE). Multichannel cuff electrode has been used to selectively activate the sciatic nerve, but these electrodes are found incapable of achieving reliable selective stimulation [7, 12]. To improve selectivity, FINE flattens the nerve to spatially distribute the fascicles and bring the stimulating contacts closer to the nerve fibres [13]. However, in electric stimulation methods, the injected current takes the path of least resistance and, therefore, the flow of current cannot be easily controlled to activate the desired stimulation target without concurrently activating the unwanted regions. On the other hand, biological tissues have uniform magnetic permeability that allows for steering the magnetic flux to create focal stimulation. Consequently, magnetic stimulation holds the potential to play an important role in the selective activation of nerves.

In this work, we study computationally the potential selectivity of an array of coils on rat sciatic nerve. The target location within the nerve is stimulated using an array of four mm-sized magnetic coils. The performance metric ‘selectivity index’ is introduced to provide a measure for the recruitment of stimulation target location of the nerve with respect to other non-targeted locations. Since biological tissue has uniform relative magnetic permeability, the electric field induced in the tissue can be controlled at the targeted site by steering the magnetic field generated by each coil, under the assumption that the coils can be driven using independent circuits. Taking advantage of this feature of magnetic stimulation, we demonstrate in this Letter that the proposed stimulation array can achieve a higher degree of selectivity compared to traditional neurostimulators by using a ${\rm \mu m}$-resolution, heterogeneous, computational model of the rat sciatic nerve based on histological data of the nerve presented in [14].

## 2 Simulation model

**Fig. 1:**
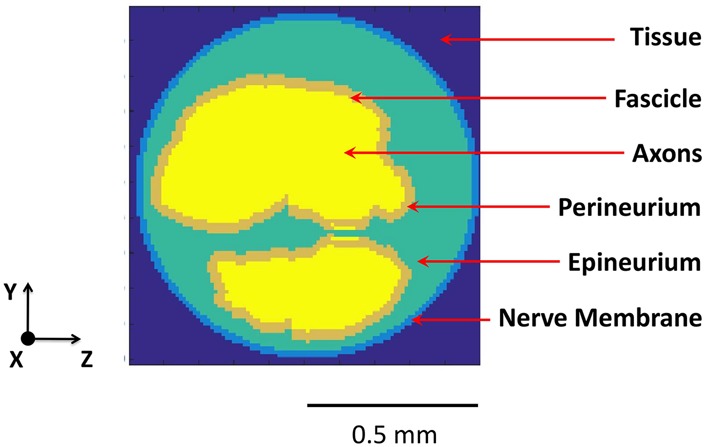
Cross-sectional view of the nerve model based on histological image of Fig. 3(D) of [14]. Various tissue types are shown with different colours: connective tissue in grey, nerve membrane (outer layer of the epineurium) in blue, epineurium (not including the outer layer) in light green, perineurium in brown, fascicles in yellow. The fascicles are populated with myelinated axons having a distribution of diameters reported in [14]

The rat's multifascicular sciatic nerve is highly structured and consists of different tissue types, such as axons, perineurium, epineurium and surrounding tissues. The different material properties of these tissue types are taken into account by constructing a fine resolution heterogeneous model. We selected the histological image of the right sciatic nerve of older rats from [14] and created a multi-fascicular nerve model to investigate the selectivity within and between fascicles. The cross-sectional view of the nerve model is shown in Fig. 1 with the orientation of *x*-, *y*-, and *z*-axis on the lower left corner. The *x*-direction is along the long axis of the nerve, and the *y*- and *z*- directions are the cross-sectional axes along the short dimension and the long dimension of the fascicles in the nerve, respectively. The nerve consists of two fascicles: tibial fascicle (the larger fascicle, upper fascicle in Fig. 1) and peroneal fascicle (the smaller fascicle, lower fascicle in Fig. 1). The simulated three-dimensional (3D) model has total size of 11 mm × 10 mm × 10 mm and the modelled nerve is 11 mm long with a diameter of 1 mm. As fine details of the structure are on the order of micrometres, the resolution of the numerical model is selected to be 10 µm along the cross-sectional *y*- and *z*-axis. Where, the resolution along the *x*-axis (i.e. along the length of the nerve) is selected as 1 mm, which is on the order of the distance between Nodes of Ranvier. To reduce the computational cost, the multi-resolution approach is utilised to minimise the number of voxels in the simulation space without introducing any approximation error at the boundaries of the model [4]. As magnetic stimulation preferentially stimulates large myelinated axons [4], the fascicles are populated with the myelinated axons having a distribution of diameters reported in the literature [14].

**Fig. 2:**
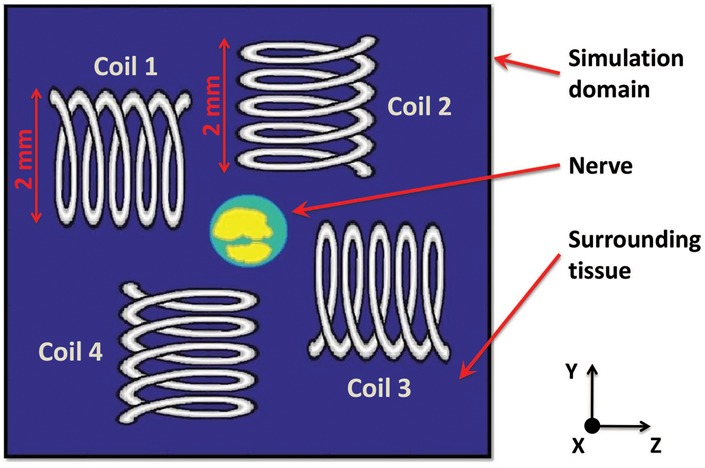
Schematic representation of the cross-sectional view of the nerve surrounded by an array of four coils. The coil-winding axis of all coils is located at the x-axis mid-point of the simulated nerve. The cross-sectional simulation domain boundaries are farther than shown in the diagram

To selectively stimulate axons in the nerve, an array of four identical mm-sized coils is used (Fig. 2). Each coil is solenoidal in shape with two layers, five turns, length of 2 mm, inner diameter of 1.4 mm, outer diameter of 2 mm, and is constructed with 32 AWG copper wire. The naming of the coils with respect to the fascicles, as represented in Fig. 2, is used throughout this Letter. The coil-winding axis is parallel to either the *y*-axis (coils 2 and 4) or *z*-axis (coils 1 and 3), and is located in the plane of the *x*-axis mid-point of the nerve. The coil-winding axis of each coil is offset from the centre of the nerve so as to place the mid-point between the inner and outer diameters of the coil closest to the nerve, which results in the largest induced electric field occurring in the nerve (explained in the Section 3) [15]. The minimum distance between each coil and the nerve is 0.5 mm.

The sciatic nerve provides sensation to most of the lower leg and skin of the foot, and innervates muscles of the lower leg and foot. For the demonstration of the selective activation, we have chosen three target regions in the nerve (as shown in Fig. 7*a*) where the motor fibres innervate: medial gastrocnemius (MG), plantaris (PL), and tibialis anterior (TA) muscles. The MG and PL muscles are innervated by the tibial fascicle, and TA muscle is innervated by the peroneal fascicle.

## 3 Theory of operation and methods

Magnetic neural stimulation is based on Faraday's law of induction, which states that a time-varying magnetic field results in an induced electric field

(1) $$\nabla \times {\bi E}\lpar {\bi r}\comma \; t\rpar = - \displaystyle{{\partial \lpar \mu \lpar {\bi r}\rpar {\bi H}\lpar {\bi r}\comma \; t\rpar \rpar } \over {\partial t}} = - \displaystyle{{\partial \lpar \nabla \times {\bi A}\lpar {\bi r}\comma \; t\rpar \rpar } \over {\partial t}}\eqno\lpar 1\rpar $$

where ${\bi E}\lpar {\bi r}\comma \; t\rpar $ is the electric field [V/m], ${\bi H}\lpar {\bi r}\comma \; t\rpar $ is the magnetic field [A/m], ${\bi A}\lpar {\bi r}\comma \; t\rpar $ is the magnetic vector potential [V$ \cdot $s/m] at position ‘${\bi r}$’ at time ‘*t*’, and $\mu \lpar {\bi r}\rpar $ is the magnetic permeability [H/m] at position ‘${\bi r}$’. The electric field, ${\bi E}\lpar {\bi r}\comma \; t\rpar $ is induced inside the nerve when current passes through magnetic coils and results in a time-varying magnetic field, ${\bi H}\lpar {\bi r}\comma \; t\rpar $. ${\bi E}\lpar {\bi r}\comma \; t\rpar $ can be calculated as

(2) $${\bi E}\lpar {\bi r}\comma \; t\rpar = - \displaystyle{{\partial {\bi A}\lpar {\bi r}\comma \; t\rpar } \over {\partial t}} - \nabla V\lpar {\bi r}\comma \; t\rpar \eqno\lpar 2\rpar $$

where $V\lpar {\bi r}\comma \; t\rpar $ is an electric potential [V], associated with boundaries between heterogeneous media. In a homogeneous medium, the term $\nabla V\lpar {\bi r}\comma \; t\rpar $ vanishes. The induced current density, ${\bi J}\lpar {\bi r}\comma \; t\rpar $ [A/m^2^], can be calculated as

(3) $${\bi J}\lpar {\bi r}\comma \; t\rpar = \sigma \lpar {\bi r}\rpar {\bi E}\lpar {\bi r}\comma \; t\rpar \eqno\lpar 3\rpar $$

where $\sigma \lpar {\bi r}\rpar $ is the conductivity [S/m] at position ‘${\bi r}$’. The stimulation threshold for an axon depends on the temporal and spatial distribution of the gradient of the electric field, $\partial E_x/\partial x$ [V/m^2^], along the long axis of the nerve and axons (*x*-axis) [4].

**Fig. 3:**
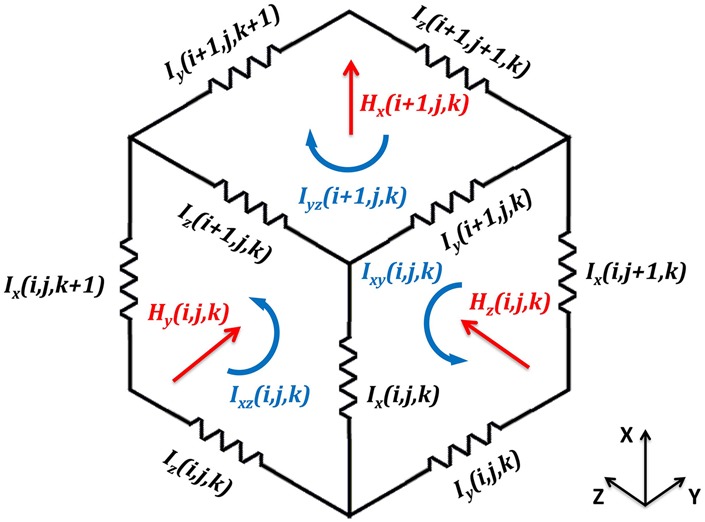
Voxel with associated magnetic field components ($H_x\comma \; H_y\comma \; H_z$), branch currents ($I_x\comma \; I_y\comma \; I_z$) and loop currents ($I_{xy}\comma \; I_{yz}\comma \; I_{xy}$). Edges of the voxel are represented by the impedances. Magnetic field components, loop currents, and branch currents are depicted in red, blue and black colours, respectively

To model the heterogeneous ${\rm \mu m}$-resolution nerve and calculate the induced electric field, we used the impedance method [16] because it solves the Faraday equation (1) in integral form and can include the effect of heterogeneity [4]. The impedance method is a frequency domain solver and it is implemented by dividing the simulation region into cuboidal voxels. Each side of a voxel is represented by an impedance value based on the electrical properties of the tissue present in the voxel (Fig. 3). In order to calculate the induced electric field in each voxel, the 3D magnetic field intensities ($H_x$, $H_y$, and $H_z$) normal to the faces of each voxel are calculated. The loop currents ($I_{xy}$, $I_{yz}$, and $I_{xz}$) at every face of the voxel are then computed from the interaction of the induced electric field on the impedances of the face. A linear set of equations are formed, using Kirchhoff's voltage law, to compute branch currents ($I_x$, $I_y$, and $I_z$) flowing through each edge of the voxel. Lastly, the induced electric field is obtained from branch currents, dimensions of the voxel, and impedance values of each cell. The temporal distribution of the induced electric field can be calculated by combining the effect of the electric field contribution at different frequencies. For the RLC stimulator circuit assumed to drive each coil, the damped frequency of the current through each coil is 2 kHz and we assumed this is the operating frequency in the simulation model.

Previously reported conductivity values of tissues are used to parameterise the model (Table 1) [4]. The nerve membrane and perineurium have higher resistivity compared to epineurium and tissue surrounding nerves. As the axons in the fascicles are oriented along the *x*-axis, the fascicles have anisotropic conductivity with a higher longitudinal value ($\sigma _x$) compared to transversal values ($\sigma _y$ and $\sigma _z$). The magnetic permeability $\mu \lpar {\bi r}\rpar $ is considered uniform throughout the tissue and not dependent on ‘${\bi r}$’. The impedance values at the sides of each voxel are calculated using the material properties and the voxel size by means of the following relation:

(4) $$Z\lpar {\bi r}\rpar = \displaystyle{{L_{\rm v}} \over {\lpar \sigma \lpar {\bi r}\rpar + {\rm j}2\pi f\,{\rm \epsilon }_{\rm r}{\rm \epsilon }_0\rpar A_{\rm v}}}\eqno\lpar 4\rpar $$

where $Z\lpar {\bi r}\rpar $ is the impedance of the edge of the voxel, $\sigma \lpar {\bi r}\rpar $ is the conductivity of the tissue types (see Table 1), $A_{\rm v}$ is the cross-section area of the voxel, $L_{\rm v}$ is the length of the voxel sides, *f* is the operating frequency, ${\rm \epsilon }_0$ is the absolute permittivity, and ${\rm \epsilon }_{\rm r}$ is the relative permittivity of the medium. At the 2 kHz operating frequency, the imaginary part of the impedance is five orders of magnitude smaller than the real part across the range of conductances in the model and, therefore, we assumed only real impedance values (i.e. resistive values) in this work.

**Table 1:** Conductivity of tissue types used in the simulation model

Tissue type	Conductivity ($\sigma _x$, $\sigma _y$, $\sigma _z$), S/m
surrounding tissue	(0.5, 0.5, 0.5)
nerve membrane	(0.02, 0.02, 0.02)
epineurium	(0.1, 0.1, 0.1)
perineurium	(0.01, 0.01, 0.01)
fascicle	(0.33, 0.08, 0.08)

## 4 Field profiles

**Fig. 4:**
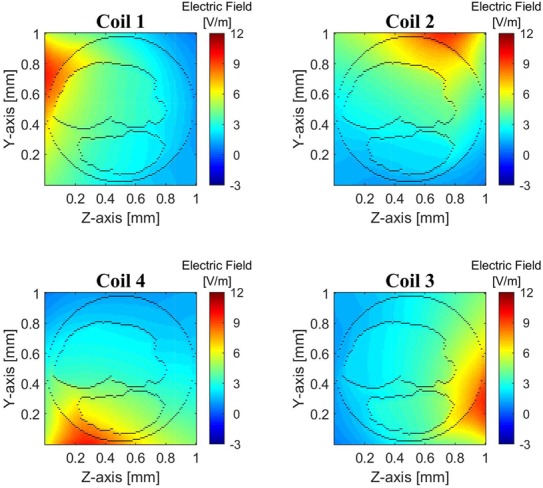
Cross-sectional view of the peak amplitude of the computed electric field induced in the nerve at its x-axis mid-point. With reference to Fig. 2, the stimulation coil number is indicated on the top of each field profile. The boundaries of fascicles and nerve are outlined by black colour

**Fig. 5:**
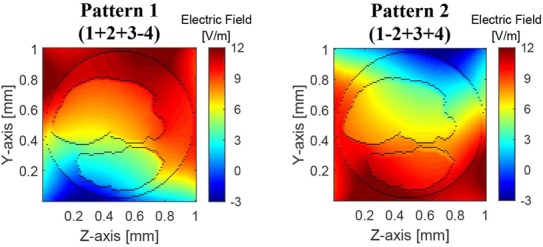
Peak amplitude of the computed electric field induced in the nerve for two different multi-coil current distributions. For Pattern 1, the currents in coils 1, 2, and 3 are in phase, and coil 4 current is $180^\circ $ out of phase. For Pattern 2, the currents in coils 1, 3, and 4 are in phase, and coil 2 current is $180^\circ $ out of phase. The boundaries of fascicles and nerve are outlined by black colour

**Fig. 6:**
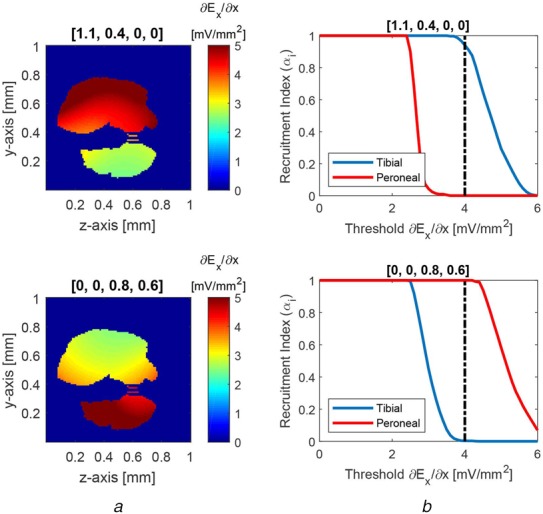
Selectivity between fascicles: upper panels represent selective activation of tibial fascicle over peroneal, and lower panels represent selective activation of peroneal fascicle over tibial. The weight vectors are shown on the top of the corresponding plots. *a* Cross-sectional view of the activation function $\partial E_x/\partial x$. To emphasise the colour map inside the fascicles, the values outside the fascicles are zeroed out for plotting *b* Recruitment of individual fascicles as a function of activation function threshold. A threshold of 4 mV/mm^2^ is used as a reference to compare selectivity indices of two fascicles

**Fig. 7:**
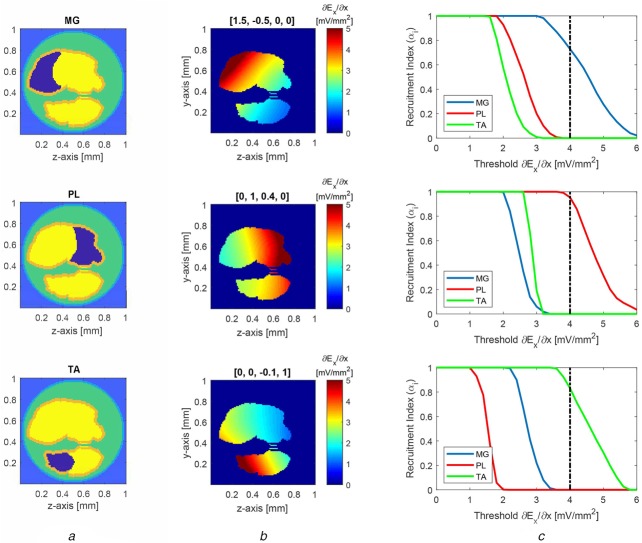
Selectivity in the fascicles: selective activation of the considered region over the other regions *a* Location of the muscular regions inside the nerve is shown in dark blue colour *b* Cross-sectional view of the activation function $\partial E_x/\partial x$ inside the fascicles. To emphasise the colour map inside the fascicles, the values outside the fascicles are zeroed out for plotting. The weight vectors used to achieve these field profiles are shown on the top of each profile *c* Recruitment of MG, PL, and TA motor neurons as a function of activation function threshold. A threshold of 4 mV/mm^2^ is used as a reference to compare selectivity indices of all three regions

Initially, we investigated the stimulation by each coil individually. The amplitude profiles of the electric field $E_x$ along the nerve, produced by each of the four coils in the simulation region, are presented in Fig. 4. All the cross-sectional slices (in *yz*-plane), plotted in Fig. 4 (and in Fig. 5, 6*a*, and Fig. 7*b*), are at the *x*-axis mid-point of the simulated nerve, which generally was the location of maximal field amplitudes. In each of the panels of Fig. 4, the coil indicated in the panel's title is driven by a 600 A peak current at the operating frequency of 2 kHz. The large current peak of 600 A is comparable to the stimulation current used in our previous work and in-vivo experiments [4, 5, 17]. The field amplitude presented in each panel of Fig. 4 is the peak amplitude of the resulting 2 kHz electric field as a function of position. Given the assumption that impedances are entirely resistive, all responses are in phase with each other. It can be observed that the electric field intensity is highest in the region closest to the driven coil, as would be expected. The boundaries between different tissue types, with the associated heterogeneity of the electrical properties, cause substantive changes in the electric field intensity at these boundaries. Further, these boundaries appear to reduce the field intensity within fascicles. A homogeneous tissue model (not shown here) fails to predict either of these effects [4].

The localised electric field profiles indicate that a limited degree of selective activation of the nerve can be achieved by individually driving each of the coils (or, the less practical option of adjusting the coil position between cases). The single coil stimulation results in the activation of only those regions of the fascicles which are near the outer surface of the nerve. To stimulate the neurons near the centre of the nerve, the coil current needs to be increased, that leads to the activation of the targeted region as well as non-targeted neighbouring regions. Therefore, to increase the effectiveness of creating strong electric fields at any location within a fascicle, we have considered the option of independently and simultaneously activating all four coils.

When all four coils are driven simultaneously, different field profiles can be induced in the nerve model; two such examples are shown in Fig. 5. The first pattern (1 + 2 + 3 − 4) represents the field profile when all four coils are driven with same unit current peak (600 A) and are in phase except for coil 4, which has $180^\circ $ out of phase current (indicated by a minus sign in front of the number 4). The second pattern (1 − 2 + 3 + 4) represents instead the field profile when the driving current in coil 2 is $180^\circ $ out of phase with respect to all other coils (indicated by −2). To compute the total field, when more than one coil is stimulated, the superposition principle is applied. That is, the field profile in the leftmost panel of Fig. 5 is the summation of all four field profiles in Fig. 4, with the field profile induced by coil 4 being negated before being added. The presence of other coils does not substantively affect the magnetic field within the nerve, which is a result of the coils being translated (see Fig. 2). To validate the applicability of the superposition principle, for a small number of cases the electric fields were computed using two approaches: (i) stimulating each coil individually, ignoring the presence of other coils and applying superposition and (ii) when all coils were present and simultaneously simulated. The electric field magnitudes computed by these two methods are found to be identical.

By driving all four coils simultaneously, larger electric field amplitudes can be observed (Fig. 5), compared to the single coil case (Fig. 4). Further, changing the sign of the coil current can result in different patterns of the electric field, which raises the possibility of steering the induced electric field by altering the relative magnitude and sign of the coil currents. To represent the relative magnitude and direction of the current flowing in a coil, a weight value is defined for each coil and the combination of the four weight values results in a weight vector. Consequently, electric field profiles can be controlled by assigning different weight vectors to the coil currents as follows:

(5) $$\lsqb I_1 I_2 I_3 I_4\rsqb = \lsqb w_1 w_2 w_3 w_4\rsqb \ast I_0\eqno\lpar 5\rpar $$

where $I_i$ is the current in the $i{\rm th}$ coil, $w_i$ is the weight assigned to the $i{\rm th}$ coil, and $I_0$ is the unit current (600 A at the frequency of 2 kHz). Herein, we only examined weight values from −1.5 to + 1.5 with negative values representing currents $180^\circ $ out of phase. Given a large number of all possible weight vectors, the optimal weight vector was selected as follows:

- The two coils farthest from the target region are assigned zero weights.
- The coil closest to the target region is assigned unity weight.
- The remaining weights are assigned values from 1.5 to −1.5 in 0.1 steps and the weight resulting in a field profile with the largest activation of the target region and least activation of untargeted regions is selected.
- The two non-zero weights were incremented up or down by 0.1 steps to maximise the selectivity index (discussed in Section 5).

As previously mentioned, the spatial distribution of the electric field gradient along the *x*-axis ($\partial E_x/\partial x$) primarily determines neural activation. This gradient is computed by numerical differentiation for each voxel of the model and subsequently compared to a voltage gradient threshold to evaluate if an axon in any particular voxel will undergo a spiking event. We used a threshold value of 4 mV/mm^2^ that resulted from a NEURON [18] simulation of a 20-${\rm \mu m}$ diameter myelinated axon in response to 1 ms stimulus pulse width. Generally, we found that the maximum gradient was observed at the *x*-axis mid-point and that we only needed to evaluate which voxels in this cross-sectional plane are within a fascicle and exceed the gradient threshold. The proportion of the voxels in the $i{\rm th}$ region that exceed the threshold is called the recruitment index ($\alpha _i$) and is defined as follows:

(6) $$\alpha _i = \displaystyle{{A_i} \over {A_{\rm T}}}\eqno\lpar 6\rpar $$

where $A_i$ is the stimulated area in the region (i.e. meets or exceeds the gradient threshold), and $A_{\rm T}$ is the total area of the region. Lastly, the selectivity indices are evaluated from the recruitment indices. The selectivity index of $i{\rm th}$ region is similar one defined in [19] and is given by

(7) $${\rm Se}{\rm l}_i = \alpha _i - \displaystyle{1 \over {n - 1}}\sum\limits_{\,j = 1\comma j - i}^n \alpha _j\eqno\lpar 7\rpar $$

where $\alpha _i$ is the recruitment of $i{\rm th}$ region and *n* is number of different regions considered. The selectivity index ranges from 1 (recruitment of the target region without the recruitment of any other region) to −1 (no recruitment of target region and complete recruitment of all other regions).

## 5 Results

First, we investigated the selectivity at the fascicular level. Using the tuning method described above, we found that we can selectively stimulate the tibial fascicle (upper panels of Fig. 6) and peroneal fascicle (lower panels of Fig. 6) through adjustment of the current amplitudes in each of the four coils [represented by the weights in (5)]. The colour maps of Fig. 6*a* present the spatial derivative of the electric field, $\partial E_x/\partial x$, for the most effective weights (values shown on the top of each panel). The weight vector of [1.1, 0.4, 0, 0] (i.e. 660, 240, 0, and 0 A peak current amplitude in coils 1, 2, 3, and 4, respectively) results in stimulation of tibial fascicle with the least stimulation of the peroneal fascicle (upper panel of Fig. 6*a*). Conversely, the weight vector of [0, 0, 0.8, 0.6] (i.e. 0, 0, 480, and 360 Ampere peak current amplitude in coils 1, 2, 3, and 4, respectively) lead to stimulation of peroneal fascicle with the least stimulation of the tibial fascicle (lower panel of Fig. 6*a*). The recruitment indices of both fascicles with respect to the voltage gradient threshold for activation are plotted in Fig. 6*b*, with the voltage gradient threshold of 4 mV/mm^2^ shown by the vertical black line. For a lower threshold (representing activation of larger diameter axons), nearly all the axons in both fascicles would be recruited and the selectivity index is almost 0. Conversely, when the threshold increases (activation of smaller diameter axons), a smaller portion of the desired fascicle is activated and the selectivity index decreases. When the threshold is increased further, neither fascicle contains any activated axons. However, one should not interpret changes in threshold as a means to selectively activate different diameter axons within a fascicle. Instead, given that magnetic stimulation preferentially activates the largest diameter myelinated axons within the region having the largest voltage gradient [4], changes in threshold represent changes in the largest diameter myelinated axons between species.

For the coil current weights, in the upper panel as shown in Fig. 6*b*, the recruitment indices are 0.95 and 0 for the tibial and peroneal fascicles, respectively, and the selectivity index is 0.95 at the reference threshold of 4 mV/mm^2^. However, if the stimulation threshold is reduced below ∼3 mV/mm^2^, both fascicles become activated and selectivity index is reduced. In order to regain the selectivity at lower threshold values, the weight vector can be scaled down linearly. Similarly, to attain the selectivity at higher threshold values, the weight vector can be linearly scaled up. In other words, the recruitment curves can be shifted left or right by scaling the weight vector, which allows for applying these results to different mammalian species with larger or smaller axon diameters.

For the coil current weights in the lower panel of Fig. 6*b*, the recruitment indices are 0.99 and 0 for the peroneal and tibial fascicles, respectively, and the selectivity index is 0.99. Again, the selectivity can be improved at other threshold values by adjusting the weight vector accordingly.

We additionally investigated the ability of the 4-coil array to selectively activate intrafascicular regions associated with different muscles (MG, PL, and TA; as shown in Fig. 7*a*) of the lower leg [6]. Using the tuning method described earlier, sets of weights were found that preferentially activate each of the three regions (three panels of Fig. 7*b*). For a gradient threshold of 4 mV/mm^2^, the selectivity indices for MG and PL, which are located in same fascicle (tibial fascicle), are 0.75 and 0.96, respectively, and the selectivity index for TA is 0.85 (Fig. 7*c*). The activation of MG muscle is more sensitive to change in the stimulation threshold, compared to PL and TA muscles. If the threshold is lowered below 3.5 mV/mm^2^, the selectivity of MG is affected as some proportion of region associated with PL also starts being activated (top panel of Fig. 7*c*). The selective activation of PL is less sensitive to the threshold and even at the lower threshold of 3.5 mV/mm^2^, PL maintains high selectivity (middle panel of Fig. 7*c*). In the case of TA, as the threshold lowers to 3.5 mV/mm^2^, part of the region associated with MG started being activated. When the stimulation threshold is raised above 4 mV/mm^2^, the selectivity of all three muscles reduces and reaches 0 eventually. Nevertheless, the selective stimulation of all three regions shows good robustness with respect to stimulation threshold, as the selectivity index of for each region remains 0.5 or better, even if the stimulation threshold is decreased (or increased) by 25%.

Further, the result suggests that the proposed stimulation method is capable of achieving higher selectivity than extraneural cuff electrodes. As reported in [20], the selectivity index achieved by cuff electrode is 0.65 for TA and 0.45 for MG, with this reference using the same definition of selectivity used herein. These values are lower compared to the selectivity index (0.85 for TA and 0.75 for MG) found in our work. In [20], the selectivity of PL is not provided, however, in our study, PL has the highest selectivity index as 0.96.

## 6 Discussion

Peripheral nerve stimulation is used for the treatment of multiple diseases including chronic migraine, facial pain, epilepsy and treatment-resistant depression [21]. Moreover, peripheral nerve stimulation can be interfaced with prostheses to restore sensory or motor functionality which is lost by a disease or an injury. For example, stimulation of the peripheral nerve can be utilised to provide the ability to control limb in an individual with paralysis [22] or to provide sensation in an individual with amputation [23]. Inside a nerve, the motor nerve fibres are grouped together based on their end connection with various muscles. Selective activation of the group of nerve fibres associated with muscles is the key behind the performance of complex muscular movements. Magnetic stimulation using the single coil approach is incapable of selectively stimulating different regions in the nerve without changing the coil location. However, the multi-coil approach presented here, selectively stimulate different regions in the nerve by simply controlling the currents flowing in each of the coils. Since the magnitude of the current flowing in a coil defines the effect on the induced magnetic and electric fields in the nerve, the position of the peaks of the aggregate electric field can be controlled by selecting the magnitude and phase of the coil currents. The obtained results suggest that target regions of the nerve can be activated with a selectivity index of 0.75 or higher. The current study is performed on a computational model of the sciatic nerve of a rat, although this modelling approach can be easily extended for more complex nerve models with a larger number of fascicles, e.g. human sciatic nerve. In cases when more than one weight vector results in likely stimulation of the desired region, the weight vector could be chosen based on the least energy requirement, with the energy requirement calculated by using the following relation:

(8) $${\rm Energy} = k\sum\limits_{i = 1}^4 w_i^2 \eqno\lpar 8\rpar $$

where *k* is a constant term and $w_i$ is the $i{\rm th}$ element of weight vector. The threshold value of 4 mV/mm^2^ used in this Letter is calculated for a myelinated axon with 20 µm diameter. However, for a different threshold value, similar selectivity can be achieved by appropriate linear scaling of the weight vectors. Despite having shown high levels of selectivity, we believe even better performance is likely possible, given that in this work, the methods of optimisation does not exhaustively investigate the stimulation space (only a maximum of two coils are driven simultaneously), space is coarsely sampled, and only $0^\circ $ and $180^\circ $ phase shifts are considered. That is, considering applying current to all four coils, examining finer resolution with the current magnitude, and allowing continuous phase relationships between the four coils may further increase the observed selectivity.

## 7 Conclusions

In PNS, nerve fibres are highly structured and grouped together according to their association with different muscles. In this work, we performed a simulation study to selectively activate various regions of the rat sciatic nerve by using an array of mm-sized magnetic coils. An array of four identical mm-sized coils is exploited for magnetic stimulation of the group of nerve fibres present in the target region of the nerve. The coil current magnitude and phase were chosen to activate one particular region in the nerve while leaving other parts of the fascicle unaffected. We have achieved a selectivity index of 0.75, 0.96, and 0.85 for the three regions (associated with the different muscles: MG, PL, TA) of the rat's nerve model. The selectivity indices for all considered muscles are higher compared to the selectivity achieved by cuff electrodes (0.65 for TA and 0.45 for MG). In a similar manner, the other regions of the nerve can also be stimulated by proper selection of weight vector. The future work will involve validating these findings experimentally.
